# A Configurable Monitoring, Testing, and Diagnosis System for Electric Power Plants

**DOI:** 10.3390/s22155618

**Published:** 2022-07-27

**Authors:** Anca Albița, Dan Selișteanu

**Affiliations:** 1 Department of Automatic Control and Electronics, University of Craiova, 200585 Craiova, Romania; dan.selisteanu@edu.ucv.ro; 2 VIG IMPEX Ltd., 200129 Craiova, Romania

**Keywords:** electric signal, monitoring, fault diagnosis, electric power plant, portable system, software application

## Abstract

The specific equipment, installation and machinery infrastructure of an electric power system have always required specially designed data acquisition systems and devices to ensure their safe operation and monitoring. Besides maintenance, periodical upgrade must be ensured for these systems, to meet the current practical requirements. Monitoring, testing, and diagnosis altogether represent key activities in the development process of electric power elements. This work presents the detailed structure and implementation of a complex, configurable system which can assure efficient monitoring, testing, and diagnosis for various electric power infrastructures, with proven efficiency through a comprehensive set of experimental results obtained in real running conditions. The developed hardware and software implementation is a robust structure, optimized for acquiring a large variety of electrical signals, also providing easy and fast connection within the monitored environment. Its high level of configurability and very good price–performance ratio makes it an original and handy solution for electric power infrastructures.

## 1. Introduction

The electric power system includes a large variety of complex electrical equipment and systems through which its adequate functioning is assured. Continuous upgrading and development must be provided to these specific elements, with the aid of specific devices and applications, adequate for the new technical conditions. Static excitation installations of electric generators, power plant produced energy evacuation machinery or automatic adjustment systems for hydro generator functioning parameters are a few examples for electric power components whose development, testing and maintenance requires the above mentioned specialized instruments. Moreover, efficient monitoring, testing or diagnosis for electric power installations has represented a permanent concern from a technical as well as a scientific point of view, which has, over time, led to completing specific applications and devices to test and evaluate the performance of the systems for which they were designed.

Data acquisition systems generally assure the simultaneous monitoring and recording of several electrical or non-electrical signals, acquired through specific sensors and transducers, specific to various practical situations. Basic concepts of data acquisition systems and brief analysis of specific application examples, corresponding to various utility contexts are detailed in an increasing number of books by specific publishers (such as [1,2]). Scientific literature also studies a great variety of applications based on data acquisition systems for fields of activity such as medicine [3,4,5], automotive [6,7], communication technologies [8,9], engineering [10,11,12], agriculture [13], and many more. As an example, the work in [3] presents an advanced data acquisition system for simultaneous EEG data of up to 20 persons. The chosen solution implies acquisition of data from each person using devices characterized by low-level microsystems connected to a server that ensures real-time monitoring and data processing. The paper [13], presents a data acquisition system for real-time control of temperature and humidity parameters imposed for grain storage. An experimental platform was used to test the system.

As can be seen, depending on the area of use, the solutions for implementing such systems must be optimized according to the practical situation requirements. In that context, electrical plants and machinery represent an important applicability sphere for this type of system, to solve complex issues related to monitoring and control. Their design and implementation are also the subject of many current publications, dealing with topics like monitoring [14,15,16], control [17,18], fault diagnosis and parameter estimation [19,20,21]. The implementation description in [22] can be considered as such a specific approach. The presented application allows data acquisition for monitoring and control (SCADA), also implementing a fault identification and predictability function.

Regarding the electric power activity domain, implementing data acquisition systems able to solve real practical issues in specific installations has represented a constant scientific concern. The research in designing and implementing various monitoring and control equipment, whose features and performance are constantly improved, has materialized. Systems recording electric power systems functioning regime parameters, or hydro generator monitoring and control systems are widely analyzed in [23,24,25].

Recently, a study focused on implementing systems for measuring and real-time display of generated parameters for configuring and testing protection relays, whose results are detailed in [26]. Moreover, data monitoring and GSM support transfer of transformer station functioning parameters is studied in [27].

During the development of new equipment and installations, the evaluation of functioning parameters in stationary and transient regimes is required to validate the results in different implementation phases. In those situations, conventional methods of measuring functioning parameters are harder to use due to the large number of parameters of interest, whose time evolution is simultaneously monitored and recorded for all electrical quantities. Moreover, simultaneous measurements and analysis of the functioning parameter behaviour during short-term (tens of milliseconds) transient regimes are necessary.

Installing and configuring specific electric power equipment and devices implies real-time evaluation of functioning parameters, during various phases of optimization and adjustment in stationary and transient regimes, prior to the completion of these works. At the end, a functioning parameter validation for the system set into operation can be performed. Gathered data during these testing activities, allow the elaboration of justifying documents regarding the functioning state of the installed equipment and framing the functional parameters of static and dynamic regimes within the limits imposed by the specifications. To exemplify, many hydro generators in the electric power plants from Romania include in their structure static excitation systems [28]. Thus, a customised structure can be optimised for the testing and configuration activities to fit the specific characteristics of these systems. Using a complex, general purpose, monitoring system to evaluate the static excitation system performances can be difficult and time consuming.

Furthermore, unexpected faults can occur in the control systems afferent to hydro generator functioning parameters (such as speed, power, etc.). In this case, the hydro generator protection system manages to isolate the fault, cancelling the possibility of performing measurements to identify the cause of the fault. A system able to perform measurements synchronous with the occurred fault would prove efficient in this case. Recording afferent parameters during the entire fault duration, including the sequence of the structure statuses generated by the automation and protection systems from the fault initiation moment until its isolation would provide substantial information to identify the fault itself. By analysing the recording, the cause generating the fault is rapidly identified, a process also known as diagnosis. Hence, the time required for repairing accidental faults is considerably shortened, outlining one more reason for implementing a data acquisition application for testing, analysing and diagnosis for electric power systems.

An available database with periodical recordings of a hydro generator’s functioning parameters is of great use to evaluate the proper operation of its functioning regimes. Monitoring and evaluation for long-term operating regimes of power generating groups afferent to electric power plants can also be the target of such applications. In addition, faults can be identified in electric power structures such as generator speed and generated voltage frequency control systems, using data acquisition systems for monitoring and diagnosis.

Portable, existent data acquisition and monitoring systems or devices cannot satisfy all these requirements and in addition, have a considerably high price. Moreover, no development intervention can be performed on these structures. As a result, local industry requests impose the design and development of a new structure, able to satisfy a sufficient requirement assembly, at maximum performances and minimum costs.

This paper describes the design and development process of such a system, and its structure is as follows. In Section 2, a concluding set of requirements is justified and established for the implemented system. The hardware structure and its specifications will be presented in Section 3. A detailed description and analysis of the afferent software suite, specially implemented for this system, is detailed in Section 4. Section 5 presents a relevant selection of various test recordings, performed with the portable system in operation, on static excitation systems afferent to hydro generators of two electric power plants in Romania. The system’s utility, along with aspects regarding the impact, targeted beneficiaries, and limitations of the work are also highlighted in this section. Finally, in Section 6, the main benefits of using this original solution are summarized, also launching the possibility of easy further development, on various directions, according to the current trends, with technologies and means chosen for similar categories of devices.

## 2. System Requirements

The specific practical examples and shortcomings analysed within the introduction highlight an assembly of features that can be required from the proposed system. The features were established to allow the use of the designed system in all three main purposes (testing, functioning regime evaluation, fault detection) presented above. Thus, the following requirement configuration would assure enough inputs, operating modes, configuring options and level of performance to satisfy the main beneficiaries from the industry field. The collection of properties, through which the main functionalities required must be provided are established as follows:

- *Real-time acquisition and display of the values of 12 analogue inputs, alphanumeric and graphical*, quantified in corresponding electrical quantities measuring units (in most situations, voltages, and currents). Their number was set to provide enough analogue inputs for the situations in which developments of the static excitation structures occur, requiring additional monitoring channels.
- *Real-time acquisition and display for 16 digital inputs*. This relatively high number was especially established for a possible extension of the fault diagnosis domain, for example in electrical plants, where the monitored and recorded signals are often of a relay type, in a larger number.
- *Implementing special operating mode features* is a mean of assuring practical versatility.
- *Ensuring a default operating mode, with the possibility of triggering and performing recordings with a settable sample number (200,000 maximum) and a settable sample duration*. This operating mode is suitable for recording electrical phenomena with fast, as well as slow time evolution, providing the user the possibility to configure the length of the recording and the sampling time interval, according to the type of studied event.
- *Ensuring a special operating mode (S1) with the possibility of triggering and performing recordings with minimum 3000 and maximum 200,000 samples, with a pre-set 12 µs sample duration, for one pre-set analogue channel.* Providing such an operating mode allows the recording of a single analogue input, with fast value changing. In this case the 12 µs sample duration allows a satisfactory sampling rate.
- *Ensuring a special operating mode (S2) with the possibility of triggering and performing recordings with minimum 3000 and maximum 200,000 samples, with a pre-set 20 µs sample duration, for two pre-set analogue channels*. It can be used when monitoring two electrical quantities, individually, or one compared to the other. The 20µs sample duration afferent in this case is still able to provide useful information in this context.
- *Ensuring pre-event recordings.* This feature is essential in synchronizing with the transient process being monitored.
- *Providing voluntary recording stop option*. When recording time is much too long and the process of interest is already over, or has already been recorded, this feature provided to the user can be useful.
- *Performed recording save and store options.* Auto generating an afferent archive with the transferred performed recording is the most suitable way of storing the data.
- *Recording view.* A means of immediately viewing the performed recording is an essential feature that must exist in the provided software suite.
- *Stored recording conversion.* If necessary, conversion from owner format into IEEE COMTRADE standard format must be provided.

The application would implement, according to the objective set, various means of triggering the recordings, thus providing the user with the possibility of setting a configuration of parameters through which to gather, within the performed recording, information completely characterizing the physical process to be analysed. In these circumstances, the user interface must allow real-time analogue input electrical quantity and digital input status views, both alphanumerically and graphically.

A robust structure with an affordable price, but able to satisfy the above-mentioned collection of requirements is being designed. The hardware structure will be organised to operate safely in an industrial environment and to provide easy connections so that a large variety of electric power devices can be tested. The afferent software provides interactive user interfaces, with features meeting the monitoring and recording requirements for the performance tests of electric power plants.

## 3. System Architecture

The innovation of this work focuses on the developed system’s ability to simultaneously provide fault diagnosis means and real-time monitoring features, beside efficient methods for performing evaluation and performance tests for various electric power systems or components. After a thorough analysis of the requirements and a research study related to the hardware infrastructure design and functions, it has been established that several types of hardware components are necessary for the configuration of the developed system to implement an application assuring the main required features:

- 12 voltage type analogue inputs;
- 8 or 16 digital inputs;
- a minimum of 14-bit resolution for analogue to digital conversion;
- synchronous sampling of analogue and digital inputs;
- individual galvanic isolation for each analogue input;
- internal power supply for digital input activation;
- external event recording synchronization circuit, provided through a tilting contact type digital output;
- serial communication interface (57,600 bauds) with galvanic isolation for RS232 serial cable connection of the data acquisition unit to a computing unit running the user software suite.

**Figure 1:**
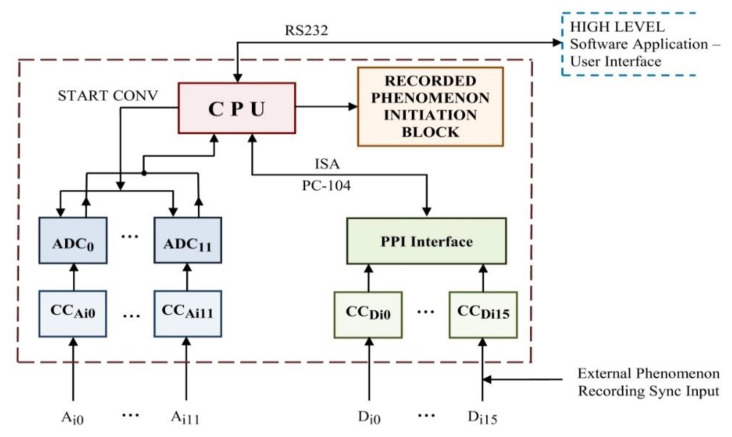
Hardware structure elaborated for the required system.

An associated diagram for a hardware structure of the acquisition unit was elaborated and it is presented in Figure 1. According to this approach, the system would include an acquisition component with an additional signal interfacing block managed by a high-level control unit, which can be represented by a laptop (possibly industrial) on which the user would manage the recording options and processes through a specially developed software application previously installed.

The data acquisition unit is equipped with a central processing unit (CPU) which initiates input signal value acquisition and enables the transfer of the minimum processed data to the computing unit from the higher hierarchical level. A “single board computer” solution was chosen for the CPU, represented by a VDX-6354 unit, with PC-104 standard, designed for an industrial environment and having an extended range of operating temperatures. The computing unit runs an associated software suite assuring the user interface and being able to interact with the firmware developed on the CPU.

To minimize the development costs, several hardware modules were implemented to assure specific functions. At the conditioning circuit level (CC_Ai_ from Figure 1) the electrical compatibility of the *analogue input signals* connected to this equipment and the analogue to digital converter voltage levels are established. This structure also assures:

- power surge protection;
- impedance adjustment and the assurance of a corresponding amplifying factor for the conditioned signal;
- input and output galvanic isolation.

The analogue to digital conversion for acquired analogue signals is performed using an analogue to digital converter for each channel, thus allowing synchronous analogue input sampling. Unlike multiplexed sampling, which implies successive sampling of each analogue channel and requiring each time the duration for analogue to digital conversion and acquiring the result by CPU, in this manner the sampling is performed simultaneously, saving (12 − 1) × the necessary time for analogue to digital conversion. Cascading 3 AD4SYNCR interface modules with synchronous sampling will accomplish the desired solution of synchronous analogue to digital sampling for 12 input channels.

*Reading the digital input state* is performed through a collection of 8-bit ports located at the PPI (parallel peripheral interface) level (Figure 1). The electrical compatibility and galvanic isolation of digital input signals is assured, as in the case of analogue inputs, using corresponding conditioning circuits, optocoupler implemented.

Within the implemented system, the high-level software application controls, through the acquisition unit implemented firmware, the diversity of running scenarios. There are two means by which the synchronizing of the recordings performed by the acquisition unit with external events is assured:

- by triggering the electric phenomenon to be recorded by the acquisition unit (temporary activation of an output contact, when starting each recording);
- by starting the recording when a digital input is activated by the infrastructure generating the phenomenon of interest, after a previous pre-recording area of data has been stored.

While developing DIAG-02, the optimization aspect was a permanent concern, as well as the low-cost requirements. As a result, a consistent research activity was allocated to finding adequate solutions for the hardware modules necessary in the implemented structure. To sum up, a short description of each main hardware component chosen or developed for the implementation of DIAG-02, and its purpose, can be found in Table 1.

**Figure 2:**
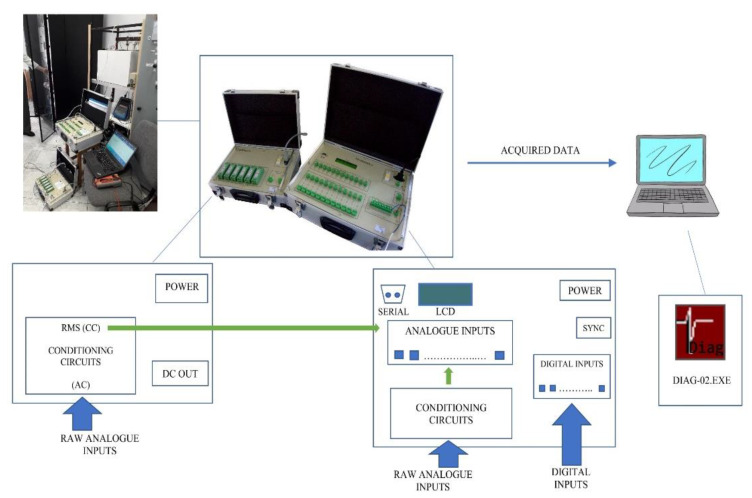
DIAG-02: Dataflow and installation example.

The newly developed data acquisition system, named DIAG-02, has a block construction, with two composing modules shielded in suitcase type covers, hence easy to transport and integrate in various electric power plant structures. Its main functions mainly consist of performing configurable recording for functioning parameters afferent to various electric power structures and performing further analyses. The system appearance, and its means of integration into a testing configuration is presented in Figure 2. Furthermore, Figure 2 highlights the connection between the system’s hardware and software components and the dataflow within its structure. According to the diagram, the electrical signals afferent to the industrial process of interest are acquired through the conditioning circuits provided by the system, with the role of signal interfacing. A low-level software application running on the acquisition device (right component of the DIAG-02 assembly) would perform and temporarily store the recording information, with the provided specifications in the recording triggering command. This command is given by the user, through the means provided by a high-level software application with a graphical interface (DIAG-02.exe), running on a computer serially connected with the acquisition device.

When recording transfer is requested, the data acquired from the connected electric power infrastructure parameters is stored in the auto-generated archive on the computer, as the destination for further analysis and interpretation.

The DIAG-02 assembly can be easily transported in the proximity of the electric cabinet from where the signals of interest are connected, and the communication with the PC is assured solely by the available serial connection. Moreover, the connections of the digital and analogue inputs to the system are made through detachable connection terminals, which can preserve intermediary connections for performing the same types of tests in a different location. All those additional features make DIAG-02 a handy portable device, optimised for various field-testing activities, diagnoses, and evaluations.

Regarding the interfacing of the analogue and digital inputs with the acquisition unit, three types of signal adjustments must be considered. In the case of continuous voltages, CC-U/U conditioning circuits convert the expected ±350 V domain for the process acquired voltage into ±10 V domain, compatible with the base acquisition block inputs (CC_Ai_ level). As it is expected that the monitored process afferent input signals of current type will be provided through Hall effect transducers, CC-I/U conditioning circuits convert the expected domain of ±100 mA into ±10 V (both CC-U/U and CC-U/I type conditioning circuits are implemented within the acquisition unit conditioning circuit block–the cover of the right DIAG-02 component from Figure 2).

Meanwhile, rms values of alternative currents and voltages are sometimes of interest, hence the existence of CC–RMS/U and CC-RMS/I type conditioning circuits must be taken into consideration. CC-RMS/U computes the rms value for an alternative voltage between 0–150 V, while CC-RMS/I finds the rms value for an alternative current between 0–20 mA. Both CC-RMS/U and CC-RMS/I convert the rms value to a value between 0 V and 10 V. The digital inputs are acquired in the data acquisition system through optocouplers. Afferent input diode polarization is performed for a 24 V voltage level.

## 4. Software Application Suite

**Figure 3:**
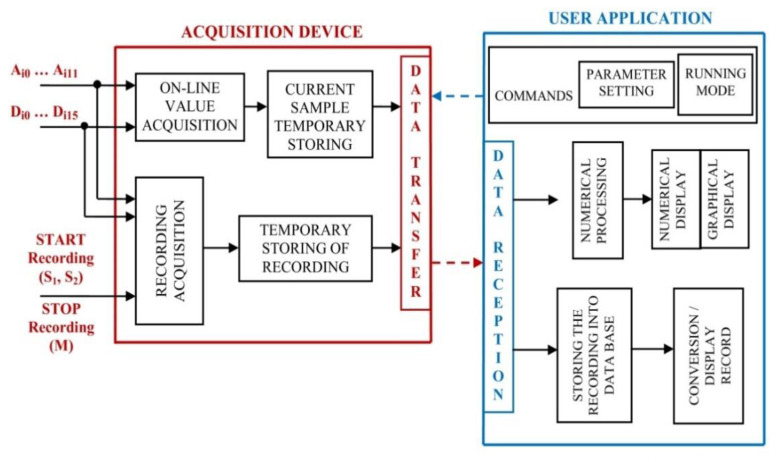
High-level/low-level interaction flow.

The functions implemented through the hardware structure are configured, commanded, and controlled during operation with the aid of a software infrastructure. The information exchange between the data acquisition unit (low-level) and the computing unit (high-level), where commands are initiated, is presented in Figure 3.

It is necessary for the software provided to assure full compatibility with the hardware infrastructure, assuring the system’s functioning stability and providing the user with all the necessary means for optimized exploitation of the configuration. As efficient use of resources was a target, even from the implementation of the hardware support, implementing a software application suite in a basic, simple approach has been the target even when establishing the main development directions.

Research in the field and implementation experience led to the objective of providing a set of applications, requiring minimum running resources:

- a firmware, managing and controlling the resources of the acquisition unit;
- a high-level user interface, which overviews and controls the system features;
- a viewer, to analyse the performed recordings.

This application set is implemented using the following available software resources:

- FREEDOS operating system for data acquisition unit’s CPU;
- Windows operating system for high-level computer, where afferent software application will be running.

The components of this software suite are exclusively implemented for this system, from firmware to user application level. The firmware is implemented according to the hardware structure of the data acquisition unit, also considering the high-level software application compatibility. Thus, the development of the software components is carried out in parallel. Meanwhile, various operating system versions existing on the high-level computer, where the user interface would be running, must also be taken into consideration. Therefore, a standalone operation for the high-level–low-level assembly must be assured. The development environment used must provide the means of encapsulating the high-level user interface resources in an application, allowing a maximum running and interaction speed.

As the interaction between the user commanding the recordings and setting the configurations, the high-level user interface providing the operating means, and the low-level firmware performing the tasks and exploiting the hardware resources is indeed complex, the implementation of the software assembly rigorously follows the basic interaction flow previously established.

The next step of the analysis determined the main blocks of resources that must be controlled through software methods, classified as the main information circulated between high-level and low-level. Thus, an initial view for the collection of information that must be available to the user is designed in this step. The result of the analysis outlined the components of an optimal software package, fully developed in our work to efficiently exploit the features of the presented hardware structure:

- The firmware of the data acquisition unit, a specific application developed using ANSI C programming language, with a loop running on the equipment, controlling the acquisition process, and assuring software means for temporary storage of the acquired data;
- The user interface application, running on a local server, which initiates the dialog with the firmware to trigger recordings with established parameters, stores the transferred data on a non-volatile support, assures real-time viewing of monitored signals and provides the user with various configuration options; this application with high complexity is implemented using a Visual C++ development environment;
- A specific analysis and recording conversion to standard format (IEEE COMTRADE) feature application, which can be launched from the user interface application.

Through this software suite, the control and recording process flow, and the user desired configurations and specific settings are provided, highlighting the need of both a firmware assured low-level resource control and a command and high-level feedback environment, represented by the user interface application. The data acquisition system features storing, recording and diagnosis functions, all made easily accessible by these above-mentioned software applications, implemented accordingly. The particularity and innovation in developing this assembly of applications reside in their customized implementation, only providing functions for efficiently controlling and exploiting the afferent acquisition system, implemented according to the specifications and resources provided. The optimisation of features prevents the use of unnecessary resources and maintains a more intuitive and easier operating interface, as the means of control for specialised users. Though minimal, in the firmware’s case, or more complex, for the user applications, the chosen developing environments can assure the means for creating standalone applications, running on the system on which they are installed, without any additional software.

The main interaction means between firmware and high-level application is represented by the mechanism through which the settings, commands and parameters are sent from high-level to firmware and implicitly to the hardware resources to perform these commands. The user can receive feedback related to the desired command execution status by two means:

- through the high-level graphical interface, which notifies whether the acquisition unit is executing a command, or the command has been performed and the equipment is available for another task (READY/BUSY);
- through the acquisition unit LCD display, which shows the established parameters when triggering a recording and also if the equipment is in the process of recording or not (READY or BUSY); if the recording was cancelled from the outside, the LCD will display the number of samples acquired, which will allow the specific useful recording fragment to be downloaded, when entered as a corresponding option on the graphical interface.

Therefore, a robust, simple, and stable communication protocol must be designed to assure the dialog between high-level and low-level, to ensure the information flow. In this context, a master–slave protocol proves to be the safest and most manageable approach. As a result, each high-level initiated request type dialog will end with returning a firmware implemented answer, corresponding to the emitted data. This answer usually contains, for a maximum of efficiency, the graphical interface requested information, either referring to on-line acquired values or a transferred recording data packet. If a communication interruption event occurs while transferring a recording, the high-level application will display an error message and the transfer of the last request packet can be resumed, on user initiative.

### 4.1. Low-Level Software Application

#### 4.1.1. Overview

The acquisition unit afferent firmware was developed with ANSI C++ to cover the following features:

- analogue and digital signal acquisition, according to established parameters;
- high-level software communication, as the provider of commands and necessary parameters for performing the recordings;
- the interpretation of external commands (of synchronizing with monitored events).

The need for permanent running of the firmware is important to mention, constantly waiting for high-level commands. As a result, the software infrastructure will be encapsulated into a permanent loop, repeating a functioning scenario until a possible stop command. In this context, an easy and stable means of implementation and integration into the final application for each functionality has been established.

According to the features provided, several main connected software modules are differentiated, each managing a function collection from the same area of interest:

- low-level running environment initialization module;
- acquisition and external event synchronization module;
- communication module;
- *main()* function module, which includes all the other composing software elements.

*Running environment initialization* implies configuring the hardware and software available instruments, accessed and used while running the firmware.

Within this application, the next items were used:

- input ports–to acquire the status of digital signals, and/or allowing synchronization with external events;
- serial communication port–to assure the communication support with the high-level computing equipment;
- input/output ports–to control the analogue to digital converter (ADC) and initiate an analogue to digital conversion (within the data acquisition subsystem);
- software interrupt–used during serial communication as well as the acquisition of new values;
- three counter timers, functioning as a divider–to assure the necessary temporizations for sample acquisition during the various imposed parameter recordings, serially requested by the high-level application.

Running environment initialization, thus assumes address assignments for hardware resources (ports) as well as communication parameter settings, memory space allocation for the used variables and their initialization.

**Figure 4:**
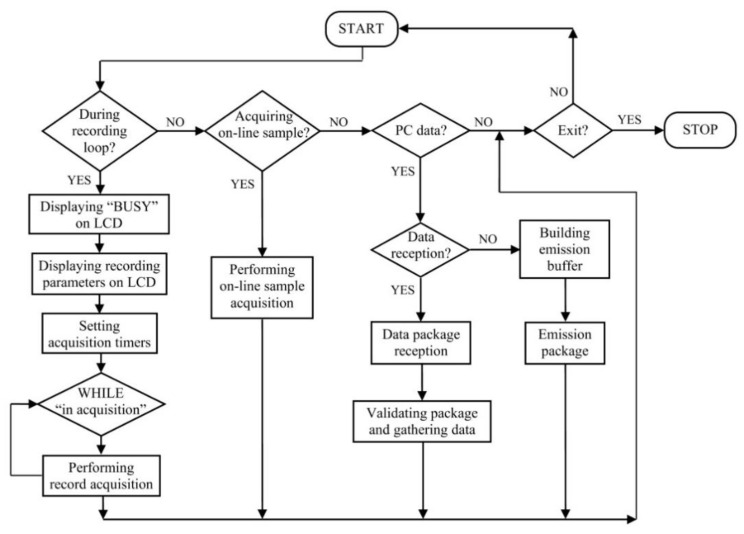
Managing the acquisition and communication interrupt.

*The data acquisition and external event synchronization module* focuses on serial communication interrupt handling. The associated interrupt function implements specialized program sequences within its functionality, as shown through the flowchart in Figure 4.

The several execution scenarios, according to the implemented features, can be distinguished:

- Online real-time acquisition sequence–assumes performing the acquisition of a single sample, at corresponding time label (after 1000 interrupts). For this sampling, an analogue to digital synchronous conversion is triggered, followed by reading and storing each channel’s acquisition result in an online sample corresponding buffer, after waiting enough time for the acquisition to have been performed; after the data transfer, the value is displayed on the user interface, on the high-level application’s initiative;
- Recording event data acquisition sequence–manages sample acquisition according to a time interval parameter on which this acquisition is performed; the acquisition is looped until gathering the user established sample number, submitted as a parameter through the high-level application options; the acquisition function establishes the time interval for acquiring a new sample, through time delays, corresponding to the time parameter value; the acquisition loop completion is signalled through a flag, tested within the communication and acquisition interrupt function;
- Communication sequence–implies the implementation of the emission–reception communication protocol, complementary to the request protocol implemented within the high-level application, for the corresponding options; the difference between emission and reception is established by testing the serial port; if bytes are received, they will be stored in a reception buffer whose content will be previously tested; the validity of a packet assumes the compliance with the emission protocol of the expected data packet; the useful information will be further retrieved and sent as parameters to the associated program sequences; when the acquisition unit must send a packet, this will be composed according to the emission protocol expected by the high-level application and serially sent, in a loop.

#### 4.1.2. Recording and ON-LINE Acquisition: Special Operating Mode

All the system features are based on efficient data acquisition, and while the hardware configuration assures optimal architecture for acquiring the data, the firmware must provide efficiency in data management and control. Therefore, assigning specific software structures and implementing the optimal methods to carry out the commands and organise the data was the next implementation step.

Establishing time intervals for acquiring a new data sample is accomplished according to the parameters sent by the user. A timer, as a component of an input–output interface afferent to the acquisition unit hardware structure, was handled in this scope. The timer resource provides three cascading counters, independently programmed as frequency dividers, so that they can correspond to the time intervals and values set by the user on the high-level interface and submitted as parameters.

Hence, using *short* data type, three such intervals can be established:

- 1 ÷ 100, corresponding to a sampling duration consisting of the set number of 1 to 100 multiplied with 50 µs, thus resulting sampling durations with an increment step of 50 µs;
- 101 ÷ 200, afferent to an interval with an increment step of 1 ms, finally accumulating sampling durations of 1 ÷ 100 ms (converting the domain of 101 ÷ 200 into (101–100) and (200–100));
- 201 ÷ 250, corresponding to a duration of the set number (201–200) to (250–200) multiplied by 50 ms, and resulting 50 ms increment step for a recording time interval.

The domain, as well as the set number, are sent via a single short integer data type parameter, the information being further used for programming the three counters. The cascaded division of the 4 MHz quartz frequency by 1000, 2 and finally with half the number set by the user (computed by subtracting the domain dimension from the sent variable) assures 1 kHz multiplied divided frequency, the same for all modes of acquisition for recording.

A recording assumes continuous acquisition of a set number of samples, received as a parameter along the time interval associated with a new acquisition (duration of the sample). As a result, acquiring a sample implies performing a sequence of functions within a specialized mechanism included in the interrupt function. While performing a recording, the data packets sent from the high-level application can no longer be received, the application entering a BUSY state. It will revert to READY after the recording is completed.

The acquisition process for a sample containing 12 analogue input values and 16 digital inputs covers the following steps:

- successively gathering the values from each analogue channel and storing appropriately in the recording buffer;
- acquiring the digital input status and storing in the same buffer;
- performing the afferent delay corresponding to the received parameter;
- testing the recording trigger bit for S1, S2, or default running modes, or the recording stop bit, depending on the case.

After the recording buffer is completed, it will be prepared for external storage from the high-level system, when the corresponding command is received.

**Figure 5:**
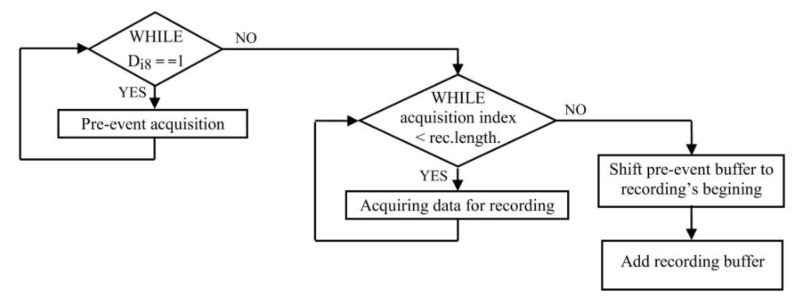
Recording with pre-event sequence flowchart.

For on-line acquisition, the analogue and digital acquired values will be saved in a current sample buffer and sent to the high-level application from 1000 to 1000 interrupts, when the acquisition unit is not recording data. The functioning algorithm for operating modes S1 and S2, mentioned in the introduction, allows the connection of a digital input to a synchronization signal, which could represent the triggering of a certain event, hence generating automatic recording with the monitoring of a pre-event sequence. The mechanism is functioning according to Figure 5, recording the pre-event in a loop of 3000 samples while waiting for the expected event to trigger, so that the final recording buffer can be completed.

### 4.2. High-Level Software Application

On the high-level computer, an application was designed to monitor and control the portable system implemented. The application also allows the start of recordings with pre-established parameters, as well as their storage in an afferent archive. Considering the features associated with these global functionalities and the specific requirements, the following set of tasks to be accomplished were established for the high-level application:

- to offer the possibility of configuring the parameters, according to the running environment;
- to provide a real-time numerical and graphical display of the monitored analogue and digital input values is necessary;
- to provide a proper variety of settings, so it can satisfy the sphere of parameter configurations imposed for commanded recordings;
- to allow the triggering and transfer of recordings;
- to assure the owner format recording storage;
- to provide a means of viewing and analysing the stored recordings;
- the option of converting the recording into standardized format for viewing must also be assured;
- to implement special operating modes, as presented in the introduction and must be implemented according to the established specifications;
- a stable serial connection must be provided;
- an easy to manipulate user interface, where all the features can be easily accessed, must be designed.

**Figure 6:**
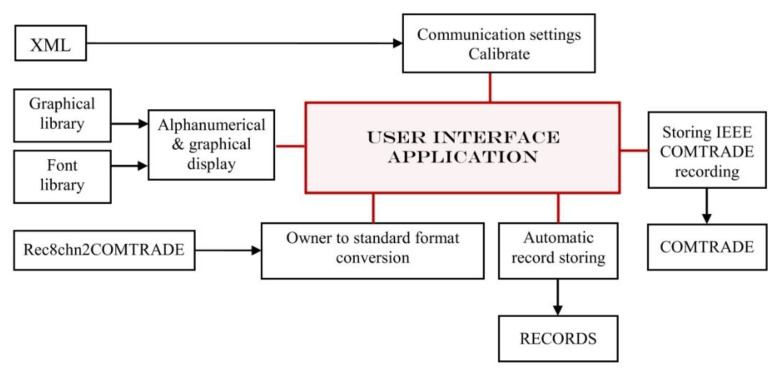
Interconnection of user interface resources.

As a command initiator and a central node of data acquisition unit provided information, various software resources and specific exploiting software mechanisms were used for the design and implementation of this complex application. The good performance of the proposed tasks assumed, according to Figure 6, management and implementation for each one through specific methods and instruments.

Analysis concluded that using graphical design functions provided by the development environment to shape the appearance of the graphical interface, with minimal external library aid, was an optimal solution, avoiding the load of additional graphical resources that could increase the delays in running, on some systems. Also, using a different standalone viewer application to analyse the recordings eliminates other software loading delays.

To sum up, the following components were integrated:

- XML file and afferent running function library, allowing easy configuration of the system from high-level point of view and possible modification at any occurring change; for this application type, the XML configuration file mainly includes communication related settings and input signal calibration;
- A complex, but suggestive and intuitive display of relevant information on the graphical interface required the features offered through a graphical library with a sufficient amount of drawing and design functions (for graphical element display) and a font library (for alphanumeric display);
- REC2chn2COMTRADE.exe, an additional application, was used while implementing the acquisition unit generated recording file conversion into binary IEEE COMTRADE files, as standard format;
- The storage of owner format recording files is performed by default in RECORDS folder, as part of its resources; when downloading is requested within the application, the files are saved under a default name;
- If an IEEE COMTRADE recording conversion is requested for an opened recording file, through the REC2chn2COMTRADE features, the resulting converted file version will be stored in a corresponding COMTRADE folder.

**Figure 7:**
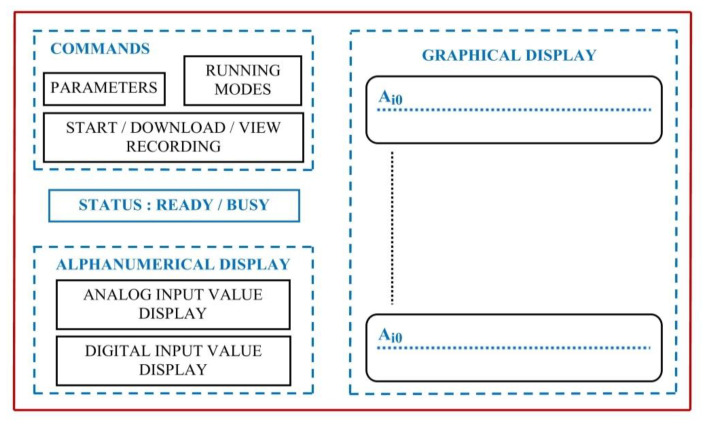
High-level graphical interface structure.

**Figure 8:**
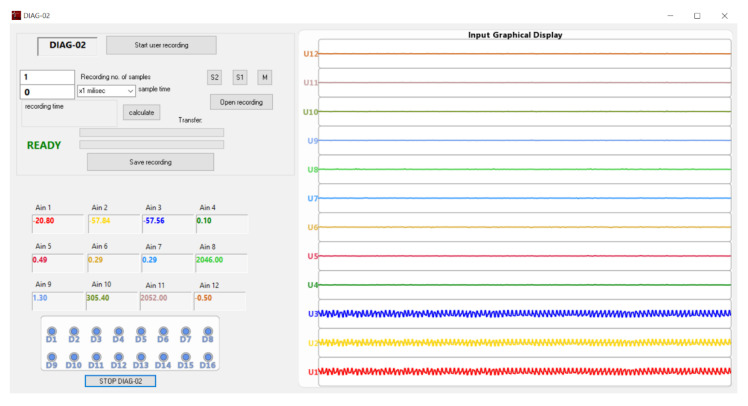
DIAG-02 user interface.

To implement this whole Windows compatible application, the developing environment used for assuring the integration and management of all external resources is Visual Studio/C++. Through it, a framework for further development as well as the needed testing and debugging tools during the implementation was assured. After detailed study of all the gathered information and a global analysis, the most intuitive way of providing the user with the configuration options, the available control means and the interaction with additional software structures has reached a final form, presented in Figure 7. The resulting user interface is presented in Figure 8.

The display gathers a command area (with operating mode selection, starting or transferring a recording) and the afferent recording parameter setting options (length, domain, sampling duration according to the selected time domain). The READY/BUSY state notifies the user whether operating on the interface is, or is not allowed, providing by default the information regarding the processing of the previous command (the BUSY message has a correspondent on the LCD display of the acquisition unit, as well as the READY message). The alphanumeric display of analogue and digital inputs is placed on the left side of the interface main frame, providing on the right side the display of a graph assembly, built based on real-time acquiring of associated analogue signal values.

From the implementation point of view, the designed resource has an associated *graphical interface class*, thus including all the functions, variables, and specific methods. The class also makes use of the features the *graphical* and *font* libraries provide. The main mean of interacting with the defined or imported auxiliary resources is the declaration of afferent objects as external resources in the interface class. Hence, its structure includes:

- serial communication parameter storing variables;
- communication class object pointer;
- application setting class object pointer;
- main window element objects;
- graphical class object;
- font type objects;
- various functions and software mechanisms for extracting interface data associated variables;
- buffer variables for acquiring current sample or transferring recordings;
- various constants associated with the communication protocol, establishing proper memory space for buffers, or distinguishing between statuses and operating modes the application runs accordingly.

**Figure 9:**
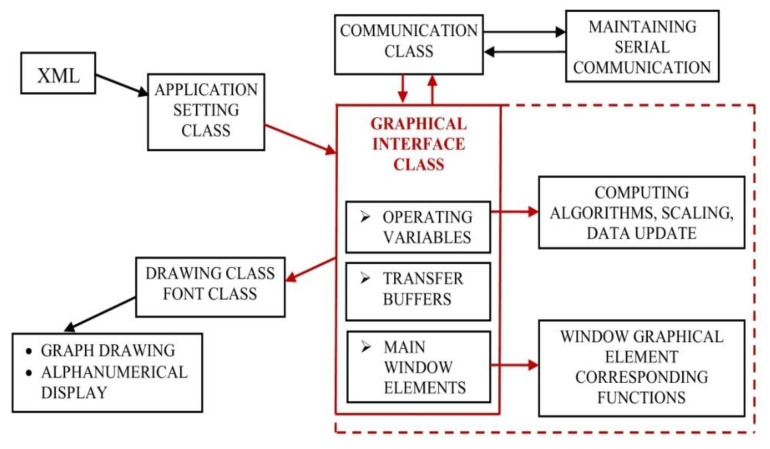
High-level graphical interface components and management.

A schematic representation of the components managed by the graphical interface class object is presented in Figure 9.

The Graphical Interface class stores the running environment configuration the user has made in the XML file through a Settings class, to manage the read information and the functions through which this reading is performed. The afferent commands and parameters are sent to the acquisition unit through the elements included in a communication class, implemented in a manner that allows the transmission and reception of a data packet collection varied enough to cover the whole functionality of the built system. The stored, scaled, and processed data is graphically and numerically displayed through the features of the graphical class and the font class used.

For an intuitive notification and graphical element view, on the user interface, the functions and properties associated with a CGraphics object were used. In addition, a CFonts object, providing font, dimension and colour modifying options for the control type elements on the graphical interface, allows their customization in a way considered adequate to the function or information it represents. The alphanumeric display differs from the graphical one, each being assigned a panel area which gathers the characteristic elements and modifies their value or aspect in real-time. Attaching a CFonts object to the static control type element was also used for displaying READY/BUSY status, which allows, or not, the user to perform settings on the interface. In this case, the default dimension and colour of the text was modified, for easier monitoring this visual component.

For the digital display area, the eight input statuses were graphically represented as LEDs, coloured in blue for zero logical and red for one logical. From the alphanumeric display point of view, each analogue input displayed on the alphanumerical panel is assigned a colour, the same being used for the graphical representation of the associated real-time waveform, on the graphical panel. The displayed data follows the configuration process algorithm, previously stored in the XML file. The graphical display is performed with each refresh of the current sample value, that is, every time the ID_ONLINE event condition is met.

### 4.3. Serial Communication

**Figure 10:**
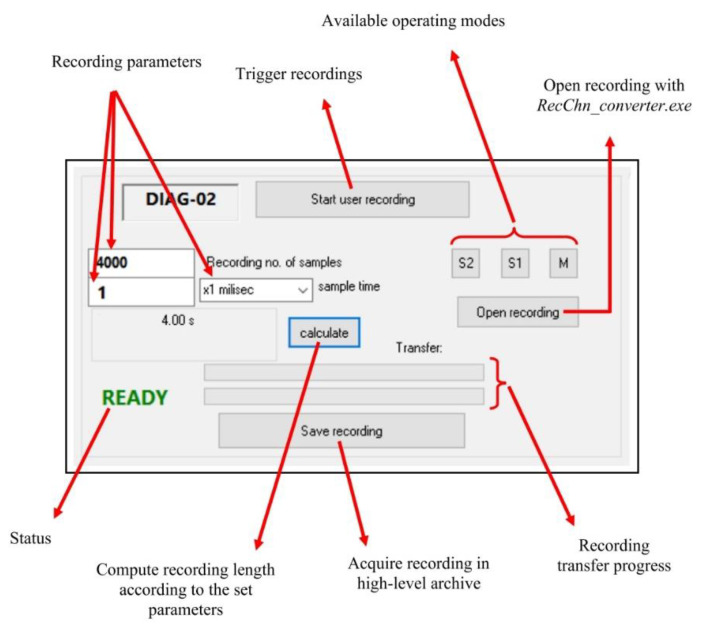
High-level graphical interface command panel.

The main available user commands, provided by the high-level application, are for triggering recordings in particular conditions and with an established parameter configuration, as shown and individually detailed in Figure 10.

The structure established for the configuring panel allows a large variety of testing condition configurations, allowing recreation of many practically met functioning situations for testing the monitored equipment. The high-level and low-level interchanged data packets have a specific owner format and a useful data order, corresponding to each information type to be sent or received. According to the implemented application, the request packet provides information regarding the command type (e.g., on-line sample request, a recording start, pre-event recording start for external event synchronization, recording transfer) and its afferent parameters.

The low-level application assigns the packet type to the corresponding data structure and format, thus preparing a response packet with the requested useful information, which can be represented by:

- the current sample buffer, for on-line value request;
- recording buffer number and offset if the loop transfer of a recording is performed.

Each request/response packet exchange respects a sequence of predefined steps:

- building the request;
- waiting for the response and resuming the transmission for a predefined number of times (established within the configuring file) in case of timeout;
- receiving the packet;
- packet validating and data extracting.

Similarly, the firmware will effectuate, for each received communication packet, validating, performing the command (or transferring the response buffer through a packet respecting the high-level format). Closing the application assumes closing the serial connection, freeing the communication pointer allocated memory and safely closing the communication handler.

## 5. Operating Mode and Results Obtained during Running in Real Electric Power Plants

### 5.1. Operating Mode

For highlighting the important features provided by DIAG-02 application and to offer a clearer view on the variety of available configuration possibilities and ways of using the designed system, significant functioning scenarios are further described.

**Figure 11:**
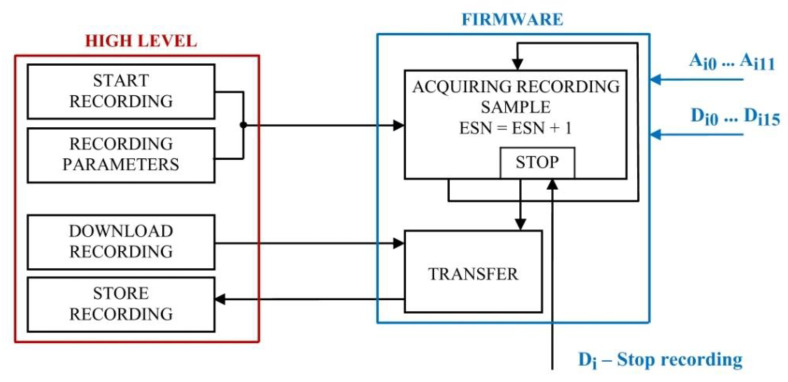
High-level/Low-level communication flow for triggering recordings.

For a proper exemplification, *a 5000-sample recording, with 1 ms sample duration is configured, with the application running in default mode (M)*. The user interface allows setting the length (Recording no. of samples) for the requested recording and the step (×1 ms), available in the associated combo-box. Regarding the communication (request/response) between the high-level application and the firmware, specific to this example and to the corresponding operating mode (*M*) is suggestively presented in Figure 11.

Retrieving the entered data will be performed when pressing the *Start user recording* button, this being stored through the corresponding software structure and prepared for transfer. This operation was included in the *GetInregParam()* function, also responsible for validating the entered data and displaying possible error messages.

When pressing the *Start user recording* button (Figure 8), a communication packet containing the requested recording parameters, set through the user interface, is sent to the firmware. In response, the firmware will trigger the recording and the user interface on high-level will enter the BUSY state. When the time interval afferent to the commanded recording ends, the user interface exits the BUSY state and enters the READY state, when the user can regain control over the interface commands. The effectuated recording can be further analysed after its conversion into IEEE COMTRADE format.

If the download of the recording is requested, pressing the *Save Recording* button (Figure 8) will initiate a high-level transfer sequence through which the request packet will send to the firmware the download index from the recording buffer and the established byte number. The response will transfer to the user application the requested information, completing the high-level recording buffer.

The dialog sequence runs in loop until the end of the transfer, the process being finalized with physically saving the delivered information, being automatically named according to a fixed format, and stored in a corresponding application subfolder.

*Performing a recording in S1 operating mode, with a length of 6000 samples and 1 × 12 μs sample duration step* is further analysed. To establish the recording length, the default 3000-sample pre-recording buffer length must be taken into consideration. Therefore, establishing an adequate length for the recording should be adjusted, so that the corresponding monitored event can be fully covered.

For this case, triggering the recording will assume two steps:

- triggering the pre-event recording, waiting for the expected event to occur, by pressing the *Start user recording* button (Figure 8) on the high-level user interface;
- triggering the actual recording, when the monitored event occurs, by activating the associated digital input; from the occurrence of this notification, the remaining recording of 6000–3000 samples will be acquired.

The transfer and view of this recording will be performed through high-level–firmware command interactions, in a similar way as the default operating mode (M). The recording will also be stored in the corresponding subfolder, under a predefined name, and be available for viewing, after a previous IEEE COMTRADE conversion, through the application provided by the user interface as well as through other specialized IEEE COMTRADE standardized viewers.

### 5.2. Real Running Condition Results

The utility and efficiency of the presented system in highlighted by a variety of experimental results obtained under real running conditions. The examples selected for this study were performed during the installation, optimization, and configuration of the functioning parameters for the static excitation installations afferent to the hydro generators at CHE Dăești and CHE Gura Lotrului from Vâlcea, Romania. During these activities, several parameter recordings, corresponding to different functioning regimes for the static excitation–automatic adjustment system–electric generator, are necessary for performance evaluation and demanding optimizations.

The view and analysis of the mentioned recordings was assured by *RECCHN_converter*, as a customized application included in the associated software suite, providing the following features of interest:

- database included DIAG-02 recording processing;
- allows viewing the analogue signal amplitude evolution in time, as well as the status of digital inputs;
- provides features for signal drawing activation of the useful parameters of an analysed phenomenon, as well as deactivating the display of unrelated elements;
- assures configuration features for signal labels, signal type (continuous or alternative), display colour, rms values, or medium values (with settable mediation factor);
- left–right display scrolling, time axis narrowing or extension, zooming the signal amplitude, significant recording area selection;
- two moving cursors and the display of values for the selection;
- printing function destined to the significant recording areas, related to user selection
- IEEE COMTRADE conversion for the processed recording, for further analysis with similar applications.

**Figure 12:**
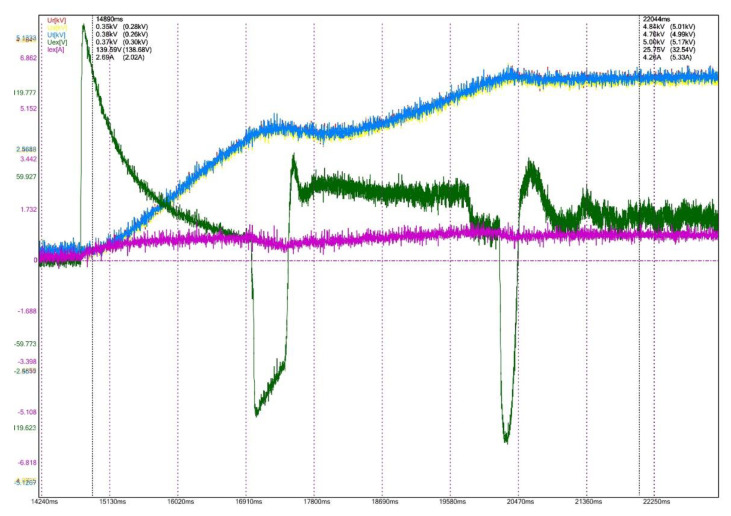
Power-on generator, CHE Dăești, Vâlcea.

**Figure 13:**
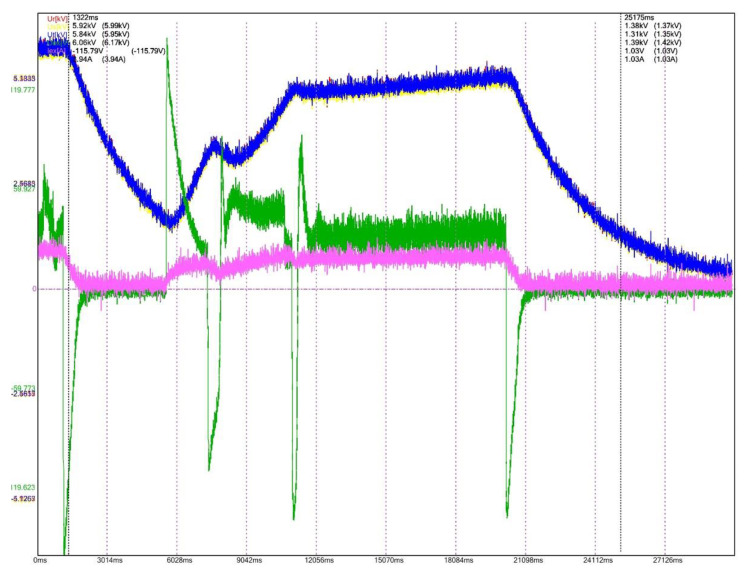
Power-off generator, CHE Dăești, Vâlcea.

**Figure 14:**
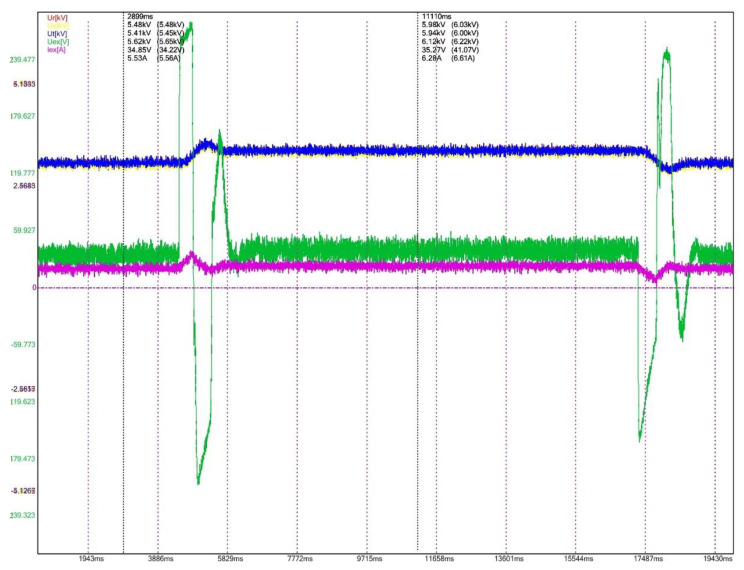
5%, 10% debited power step commands, CHE Dăești, Vâlcea.

**Figure 15:**
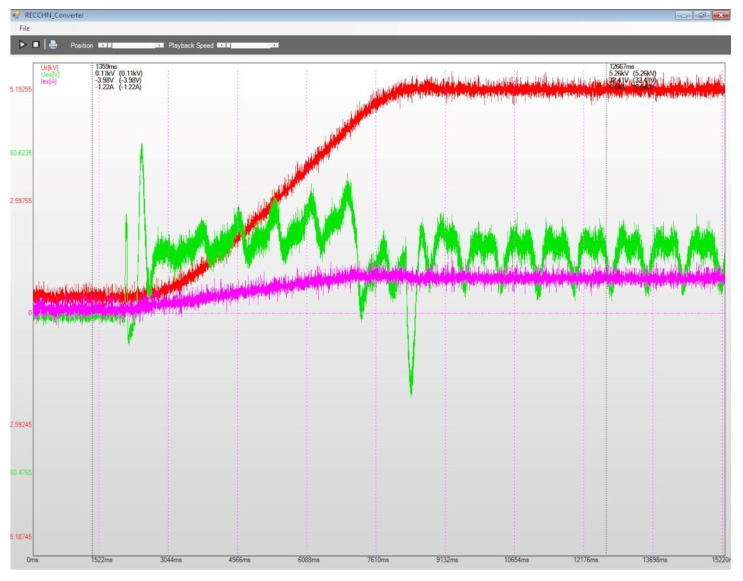
Power-on generator, CHE Gura Lotrului, Vâlcea.

**Figure 16:**
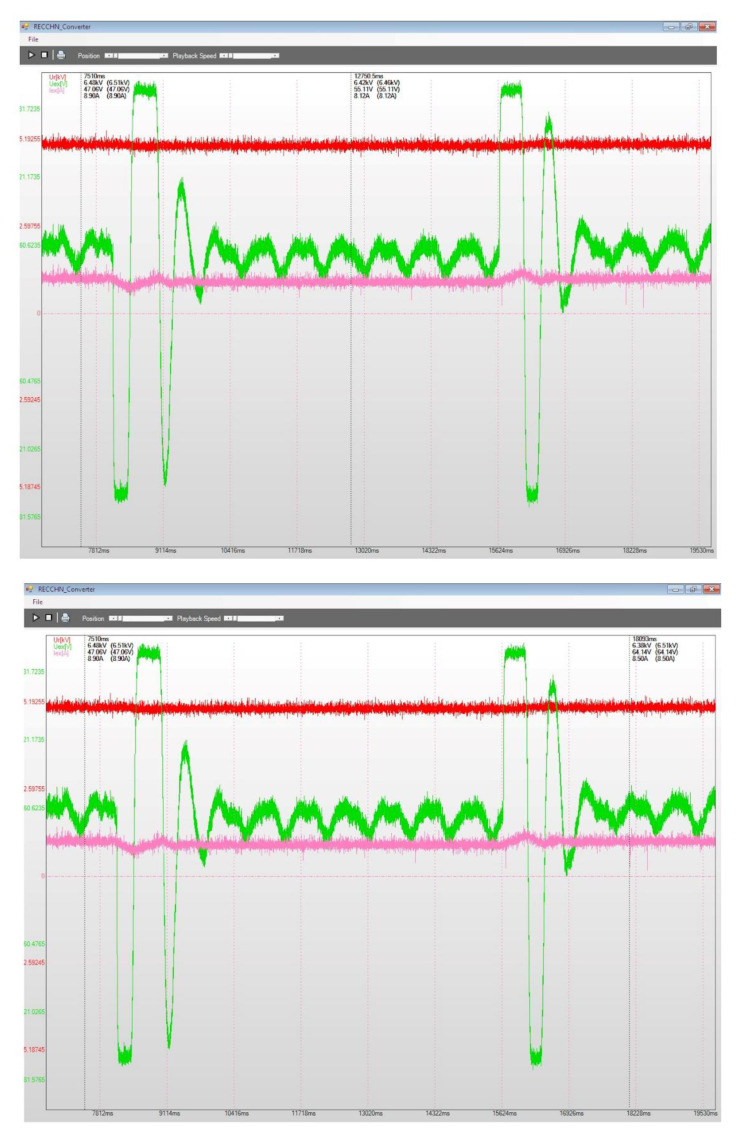
5% step power decrease (top diagram) and increase (bottom diagram), CHE Gura Lotrului, Vâlcea.

**Figure 17:**
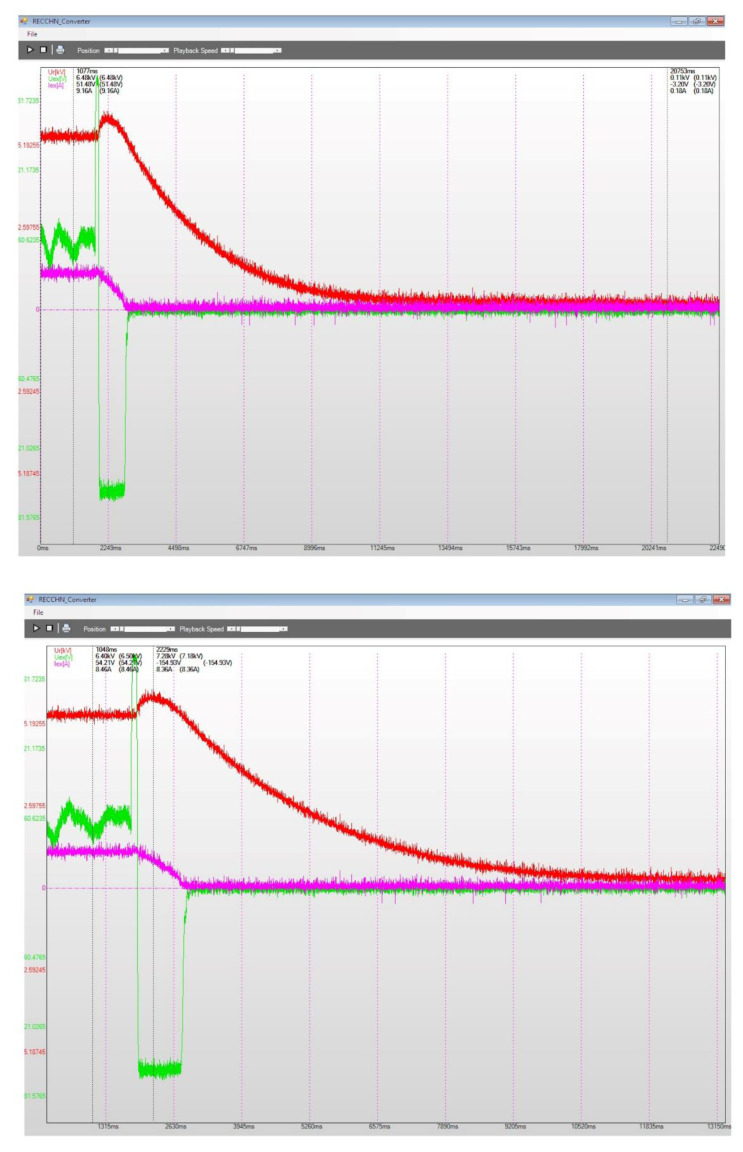
Power−off generator with electric charge throwing, CHE Gura Lotrului, Vâlcea, before and at the end of transient regime (top diagram), also before and during the transient regime (bottom diagram).

To evaluate the performances for the excitation equipment afferent to the electric generators running in hydropower plants, several electrical signals of interest, present in Figure 12, Figure 13, Figure 14, Figure 15, Figure 16 and Figure 17 are monitored and synchronously recorded, for further post-analysis.

For the presented practical situations, the significant electrical signals characterizing the assembly of *static excitation and control system–electric generator* are as follows:

- rms values of line voltages afferent to the electric generator, Ur, Us, Ut, coloured in red, yellow, and blue–overlaid in diagrams;
- electric generator excitation voltage, Uex, controlled through the automatic adjustment system afferent to the static excitation installation, coloured in green;
- electric generator excitation current, Iex, coloured in orange or pink in diagrams.

The mentioned electrical signals are acquired from the electrical process through the data acquisition system DIAG-02 by the aid of sensors, transducers, and conditioning circuits, adapted to the practical situations met in the field (all components of the hardware structure).

Therefore, the phase voltages at the electric generator terminals (Ur, Us, Ut) are gathered from the voltage reducers existing in the analysed electric installation and applied to the conditioning circuits included in the designed acquisition system. The excitation current (Iex) is acquired using a Hall effect transducer, and the excitation voltage (Uex) is applied to the corresponding conditioning circuit, from the data acquisition system structure.

Figure 12 presents the transient regime for the generator starting process, equipped with the static excitation installation whose performances are verified (CHE Dăești, Vâlcea, Romania). The time evolution of the significant electrical signals corresponding to this dynamic regime can be observed in the diagram.

During the recording process, several excitation voltage fluctuations are observed (Uex), which implicitly leads to excitation current oscillations as well. The automatic adjustment system controls this behaviour, so that after a few variations, the stabilization of generator voltages Ur, Us, Ut occurs. This type of recording allows an estimation for the generator starting time.

In a similar way, the transient event of generator shutdown command (Figure 13) can be analysed. The time behaviour of the significant electric signals (Ur, Us, Ut, Uex, Iex) is highlighted in the recording, starting with the moment of stabilized regime functioning until the generator voltages values reach 0. The Uex voltage and, in consequence, associated Iex current fluctuations also lead to variations of the generator’s voltages. After the settling of those variations, the voltages decrease to 0 value.

Figure 14 describes a performance test for the static excitation–hydro generator assembly effectuated for 5% and 10% debited power step commands. The time evolution of command signals (Uex and Iex) is analysed, also supervising the maintenance of the generator terminal voltage values into imposed limits. It can be observed that from the stabilized regime of the first cursor to the stabilized regime of the second cursor, the generator voltages are increasing, compared to the nominal operating mode.

Figure 15, Figure 16 and Figure 17 present several recordings corresponding to performance tests for the static excitation equipping a hydro generator from CHE Gura Lotrului, Romania. The afferent diagrams illustrate the time evolution for the primary values of the recorded electrical quantities.

The recording from Figure 15 allows an evaluation for the transient regime of starting the generator. Variations of the excitation voltage are observed, concurring with an increase in the excitation current, until a stable regime is obtained. It is also noted that the generator voltages (highlighted by phase voltage Ur) increase from 0 value (first cursor) to nominal value (second cursor).

Figure 16 shows the static excitation–hydro generator assembly (CHE Gura Lotrului, Vâlcea, Romania) behaviour on commanded 5% stepped power decreases and afterwards returning to the previous situation by a 5% power increase. When the power decreases, the excitation voltage has a negative jump, followed by several variations and the stability of the functioning regime (the top diagram). When the power increases by 5%, a first positive impulse is observed at the excitation voltage, followed by a few fluctuations and its transition to a stabilized regime (the bottom diagram).

Figure 17 analyses a transient regime for powering off the generator (*CHE Gura Lotrului, Vâlcea, Romania*) with electrical charge loss (practically called “electric charge throwing”). In this situation, the excitation current must be decreased to 0 during a minimum time (the moment before first cursor position, in the top diagram) so that the generator voltage has the minimum outgrowth caused by the increase in speed (second cursor position, in the bottom diagram). This precaution is managed by the control system of the static excitation installation through a negative *Uex* voltage impulse. The second cursor position from the top diagram identifies the end of the generator power off transient regime.

The DIAG-02 system was completed in April 2021 and was used for completing the performance tests of several hydro generator static excitation systems in over four locations, including the static excitation system manufacturer (ICPE ACTEL [28] and the static excitation system beneficiary (HIDROELECTRICA S.A.) after their installation, while testing a pwm waveform voltage generator (ELSSA Laboratory, Ltd. [29]).

The system versatility is also proved by the fact that it has already been used for the following purposes:

- for testing hydro generator static excitation systems, at CHE Dăești, CHE Gura Lotrului, Romania;
- for diagnosis, at CHE Ostrov, HIDROELECTRICA SA Hațeg, Romania;
- for analysing the functioning regimes of hydro generators, at CTE Turceni, CTE Rovinari, Romania.

The exemplified recordings performed during hydro generator static excitation systems post-installation tests were obtained in the second part of year 2021 and the first part of year 2022.

DIAG-02 is the most adequate mean through which the local static excitation system manufacturers can justify the performances and proper functioning of their developed work, representing a more convenient alternative to the use of multi-fault recording devices, especially in the voluntarily triggered evaluation probe field.

The detailed reproduction of the generated phenomenon, revealed in the presented triggered recordings, demonstrate the system’s efficiency in performing post-installation evaluation tests on hydro generator static excitation systems. As the structure has also already been used for fault diagnosis in several electric power plants from Râul Mare, Retezat hydropower development (CHE Ostrov included), it can be proved that the system has an important contribution in the fast identification of unexpected critical failures.

The DIAG-02 system measurements assure a better than 1% precision, more data accuracy not being necessary or requested, given the fact that the evolution of phenomena over time is of interest and concern.

### 5.3. Critical Analysis and Discussions

By reviewing the technical features and examples of use, the target beneficiaries of DIAG-02 systems can be established:

- generator afferent static excitation system manufacturers;
- electric power plants’ maintenance staff;
- controllable waveform voltage generator manufacturers;
- electric power field research teams for analysing generator functioning regimes and identifying hidden faults.

Several hundreds of electric power plants having hydro generators which need periodic revisions or static excitation system replacements exist in Romania. Carrying out these activities leads to resuming the performance tests, where the use of a portable monitoring, testing, and diagnosis system such as DIAG-02 simplifies the process. Moreover, considering the diversity of generator types whose features are covered by DIAG-02, the system can be used with possible minimal changes for testing and revision activities of corresponding systems afferent to other types of power plants (e.g., wind farm), located not only in Romania.

The spectrum of testing and diagnosis activities in which DIAG-02 is used can be assimilated to the multifunctional disturbance fault recorder devices field [30]. However, the price range for these devices is over €12,000. DIAG-02 ranges from €6000 to €8000, according to the complexity of the delivered supply (transducers, signal converters, etc.). Replacing DIAG-02 features by using several types of testing devices available on market is also not convenient for the user, both in terms of price and ease of operation.

Meanwhile, it can be highlighted that DIAG-02 is not a system suited for high precision measurement, in its area of operation the parameter evolution in time, as well as the parameter transition sequence is of interest. Also, the recording downloading speed currently limits the recording length to 200,000 samples, a restriction that is intended to be removed by replacing the RS232 communication protocol with Ethernet. A software limitation is the lack of an analysis feature development integrated in DIAG-02.exe. The software application currently assures standard IEEE COMTRADE format conversion for the recordings, for further interpretation with the aid of specialized analysing software applications, IEEE COMTRADE compatible. In exchange, a basic collection of viewing features for owner-format recordings is assured through *RECCHN_converter*, an application available in the DIAG-02 software suite.

The use of complex data acquisition and control systems (e.g., SCADA) for identifying or estimating occurring faults is a current concern and a novel approach. To exemplify, the method presented in [31], fault prognosis specific to wind turbine bearing failures uses a collection of relevant data through a set of variables of interest, provided through the afferent SCADA system. The gain of such an approach is related to an efficient use of information, as additional monitoring of specific parameters is not needed, and estimations are made based on already existing data in SCADA. Moreover, a specific data acquisition infrastructure is no longer required. Identifying wind turbine specific faults through different specific data processing strategies, already acquired through SCADA, was also approached in [32]. Through targeted analysis and associated simulations, the method has proven its efficiency and utility within condition monitoring in wind turbines.

As a parallel, from the available data analysis point of view, the DIAG-02 recording purpose is the identification of sudden unexpected faults through chronological analysis of the system-specific parameter evolution in time. It is the reason why DIAG-02 provides the fault synchronisation feature, easing the analysis and highlighting the fault cause.

Imposing a performance indicator set for offering an overview on monitored structures is an approach discussed in [33] with highlighted and demonstrated importance. Related to the present work, establishing a set of performance criteria based on the recording archive information resulting from performance tests would give the manufacturer a better view regarding the evolution and performance satisfaction degree for hydro generator designed static excitation structures and systems.

## 6. Conclusions

The portable data acquisition and diagnosis system, PC-09/DIAG-02 is designed for sampling, temporary data recording and transfer to a higher-level computing equipment for further storing and processing. The PC-09/DIAG-02 features justify its usefulness while recording electrical quantities specific to electric power installations, for the purpose of analysing their operating modes (stationary or transient) and possible diagnosis. PC-09/DIAG-02 configuring features allows it to perform recordings and analysis for short transient regimes, as well as for long-term operating regimes (hours or tens of hours).

The presented application is extremely useful for identifying untimely faults occurring in electric power installations, whose causes tend to be difficult to identify. For example, when a feedback loop is interrupted in an automatic speed control system of a hydro generator (which can be caused by temporary damage to a connection due to vibration or weakening of the pressure in a string clamp) the whole system is blocked, with impossible means of measuring the signals that can be conducive to identifying the fault. Recording the significant monitored installation signals, the situation generating the fault can be identified, by analysing the signal evolution during the transient regime generated by the fault, leading to its primary cause. In such situations, DIAG-02 can be connected within the installation as so called ‘spy’.

The practical testing requirements can be solved by using various equipment such as *analysers* [34] or *recorders* [35]. These type of devices usually require other auxiliary components (transducers, conditioning circuits) for their fitting in the tested electric power installations. Furthermore, the consumed time for connecting the assembly to the signals of interest in electric power installations and equipment should be considered. The existence of a specialized system to perform tests, similar to the mentioned examples, is preferred. Regarding hardware, such systems assure a fast placement in electric power installations and easy connection to the signals characterizing the recorded and analysed event. This approach can also reduce the implementation costs and increase the optimization of testing and diagnosis activities (at the beneficiary).

Regarding future research directions, three main development strategies can be identified to improve the functionality and performances of DIAG-02:

1. Minor hardware improvement, such as replacing serial communication with local area network communication, can be implemented. This would ensure better speed performances and less parameter limitations for the afferent software applications. Software improvements can be initiated as well as including more of the individual high-level application features into the main graphical interface application, and extending its current functions.
2. For some applications, the typical structures of data acquisition systems do not ensure adequate performance. In such situations, these structures are optimized, with resulting configurations differing from the typical implementation. Equipping each analogue to digital conversion channel with a memory block (SRAM), in addition to the afferent analogue to digital converter, can obtain highly improved signal sampling rates for a synchronous acquisition structure. Such an approach is also discussed in [36].
3. The popularity of IoT implementations in recent times raises the issue of developing systems with remote monitoring and control features, starting from the data acquisition core implemented within the DIAG-02 system. The strategy has been successfully implemented in domains such as electric power [37,38,39], medicine [40], industry, environment quality [41,42,43,44]. As the authors’ subsequent preoccupations are also oriented towards the development of data acquisition systems with remote communication features, the possibilities of implementing IoT applications and systems providing performances related to the electric power field of activity are analysed.

## Figures and Tables

**Table 1:** Main hardware components of the acquisition unit.

Functioning Block	Description and Purpose
**CPU**	Central processing unit: initiates data acquisition, assures serial communication functions, performs data transfer-VDX-6354 single board computer;
**CC_Ai_**	Analogue input signal conditioning module, designed and developed within the implementation of DIAG-02: assures ADC supported voltage levels, power surge protection and galvanic separation;
**CC_Di_**	Digital input signal conditioning module, with optocoupler, with voltage levels of logical 0 = 0 V and logical 1 = 24 V; designed and developed within the implementation of DIAG-02;
**ADC_i_**	Successive approximation digital to analogue converter, part of a synchronous sampling structure with digital multiplexing;
**Recorded phenomenon initiation block**	Event synchronization block: assures recording synchronization with the triggering of the monitored transient regime;
**AD4SYNCR**	Data acquisition interface with synchronous sampling and 4 channels; can be cascaded with more units to extend the input range; designed and developed within the implementation of DIAG-02.
